# Association of Urinary Bisphenol A Concentration with Heart Disease: Evidence from NHANES 2003/06

**DOI:** 10.1371/journal.pone.0008673

**Published:** 2010-01-13

**Authors:** David Melzer, Neil E. Rice, Ceri Lewis, William E. Henley, Tamara S. Galloway

**Affiliations:** 1 Epidemiology and Public Health Group, Peninsula Medical School, University of Exeter, Exeter, United Kingdom; 2 School of Biosciences, University of Exeter, Exeter, United Kingdom; 3 School of Mathematics and Statistics, University of Plymouth, Plymouth, United Kingdom; East Carolina University, United States of America

## Abstract

**Background:**

Bisphenol A (BPA) is a high production volume chemical widely used in food and drinks packaging. Associations have previously been reported between urinary BPA concentrations and heart disease, diabetes and liver enzymes in adult participants of the National Health and Nutrition Examination Survey (NHANES) 2003/04. We aimed to estimate associations between urinary BPA concentrations and health measures in NHANES 2005/06 and in data pooled across collection years.

**Methodology and Findings:**

A cross-sectional analysis of NHANES: subjects were n = 1455 (2003/04) and n = 1493 (2005/06) adults aged 18–74 years, representative of the general adult population of the United States. Regression models were adjusted for age, sex, race/ethnicity, education, income, smoking, BMI, waist circumference, and urinary creatinine concentration. Main outcomes were reported diagnoses of heart attack, coronary heart disease, angina and diabetes and serum liver enzyme levels. Urinary BPA concentrations in 2005/06 (geometric mean 1.79 ng/ml, 95% CI: 1.64 to 1.96) were lower than in 2003/04 (2.49 ng/ml, CI: 2.20 to 2.83, difference p-value = 0.00002). Higher BPA concentrations were associated with coronary heart disease in 2005/06 (OR per z-score increase in BPA = 1.33, 95%CI: 1.01 to 1.75, p = 0.043) and in pooled data (OR = 1.42, CI: 1.17 to 1.72, p = 0.001). Associations with diabetes did not reach significance in 2005/06, but pooled estimates remained significant (OR = 1.24, CI: 1.10 to 1.40, p = 0.001). There was no overall association with gamma glutamyl transferase concentrations, but pooled associations with alkaline phosphatase and lactate dehydrogenase remained significant.

**Conclusions:**

Higher BPA exposure, reflected in higher urinary concentrations of BPA, is consistently associated with reported heart disease in the general adult population of the USA. Studies to clarify the mechanisms of these associations are urgently needed.

## Introduction

Bisphenol A (BPA) is a man-made compound that is suspected to act as an endocrine disruptor, i.e. a compound capable of causing dysfunction to hormonally regulated body systems [1]. More than 2.2 million metric tonnes of BPA are produced worldwide each year for use mainly as a constituent monomer in polycarbonate plastics, which are used extensively in drinks containers and food packaging, and in the production of epoxy resins used in the lining of canned goods [2]. Widespread and continuous human exposure to BPA is believed to be mainly through dietary intake [3], with additional exposure through drinking water, dental sealants, dermal exposure and inhalation of household dusts. It is one of the world's highest production volume compounds and human biomonitoring data indicates that the majority (over 90%) of the general population is exposed to BPA, evidenced by the presence of measurable concentrations of metabolites in the urine of population representative samples [4], [5], [6].

The potential for BPA to cause adverse human health effects is believed to be a consequence of its well-documented estrogenic activity, with reports of both estrogen agonist [7] and androgen antagonist [8] activity. Reported additional modes of action [9] include liver damage [10], [11], [12], [13], [14], disrupted pancreatic Beta-cell function [15], thyroid hormone disruption [16] and obesity promoting effects [17]. Many of these effects have been reported to occur at concentrations below recommended safe daily exposure levels, prompting much recent debate on the requirement for revision of current legislation [18], [19], [20], [21].

Once ingested BPA is metabolised to form the highly water soluble major metabolite, bisphenol A-glucuronide. Volkel et al reported that the half life for renal clearance of this metabolite from blood following oral administration was 5.3 hours in adult male and female subjects [22]. Exposure studies in humans are restricted due to ethical reasons and by the difficulties in finding individuals completely unexposed to BPA from the environment. As such there are no *in vivo* data on the rate at which unconjugated BPA is converted to bisphenol A-glucuronide in humans, only predictions [6]. Given the urinary route of clearance of the major metabolite, urine is considered to be the most appropriate body fluid for BPA exposure assessment. Based on the animal and laboratory evidence, we previously hypothesised that higher urinary BPA concentrations would be associated with adverse human health effects, especially in the liver and in relation to insulin, cardiovascular disease and obesity. In 2008 data were released from the US National Health and Nutrition Survey (NHANES) 2003/04 providing the first large-scale population-representative epidemiological data on urinary BPA concentrations with sufficient statistical power to detect low-dose effects [6]. Higher BPA concentrations in NHANES respondents were found to be associated with cardiovascular disease diagnoses (Odds Ratio (OR) per 1 standard deviation (SD) increase in BPA concentration = 1.39, 95% CI 1.18–1.63; p = 0.001 in fully adjusted regression models). Higher BPA concentrations were also associated with diagnosed diabetes (OR per 1SD increase in BPA concentration = 1.39; 95% CI 1.21–1.60; p<0.001) but not with other common diseases [23], [24], [25],suggesting specificity of the reported findings.

These first reports clearly need to be replicated in independent study samples, to ensure that the findings are robust and to refine estimates of effect sizes. Here we report an epidemiological analysis of the NHANES 2005/06 BPA biomonitoring results, which measured BPA levels on a new independent cross-sectional sample of the US non-institutionalized population. We re-tested the originally identified associations of higher urinary BPA concentrations associated with reported heart disease, diabetes and liver enzymes. We also estimated the strength of these associations in all the available data (combining NHANES 2003/04 and 2005/06, termed here ‘the pooled data’.)

## Methods

Data were from the US NHANES study 2003/04 and 2005/06 [26]. The study assesses health and diet, and the samples are representative of the non-institutionalized population of the United States. NHANES surveys are cross-sectional, recruiting new samples for each wave. NHANES is administered by the National Center for Health Statistics, the Health Statistics Institutional Review Board of which approved the study.

### Ethics Statement

NHANES is a publicly available data set approved by the National Center for Health Statistics institutional review board, and all participants provide written informed consent.

### Assessment of Bisphenol A Concentrations

A one-third random subset of NHANES 2003/04 and 2005/06 participants supplied urine samples which were analyzed for BPA. Studies of the temporal variability in urinary BPA concentrations have reported single urinary measurements to show moderate sensitivity for predicting an individual's tertiary categorization [27]. A spot urine sample was collected from each respondent and total (free and conjugated) urinary concentrations of BPA were measured by the Division of Environmental Health Laboratory Sciences (National Center for Environmental Health, Centers for Disease Control and Prevention) using online solid-phase extraction coupled to high-performance liquid chromatography–isotope dilution tandem mass spectrometry with peak focusing. The comprehensive quality control system included reagent blanks to ensure samples were not contaminated during handling, storage and analysis. (See http://www.cdc.gov/nchs/data/nhanes/nhanes_03_04/l24eph_c_met_phenols.pdf & http://www.cdc.gov/nchs/data/nhanes/nhanes_05_06/eph_d_met_phenols_parabens.pdf [accessed 22 September 2009]. The lower limit of detection (LLOD) for BPA concentrations was 0.36 ng/ml in 2003/04 and 0.4 ng/ml in 2005/06. BPA results below the LLOD (n = 116/1455 in 2003/04 and n = 126/1493 in 2005/06 aged 18/74 years with measured BPA and urinary creatinine levels) were replaced with a value equal to the LLOD divided by the square root of two in order to distinguish between a non-detectable laboratory test result from a measured laboratory test result. We assigned the 2005/06 derived “low” value of 0.28 to all individuals from either NHANES years with BPA concentrations below the LLOD.

### Health Outcomes

Questions relating to medical conditions were asked before the physical examination, in the respondent's home, using the Computer-Assisted Personal Interviewing (CAPI) system. Respondents aged 20 years and over were asked “Has a doctor or other health professional ever told you that you have…” for angina, coronary heart disease, heart attack, stroke, asthma, emphysema, chronic bronchitis; arthritis, thyroid problems, any kind of liver condition, or cancers. We defined ‘cardiovascular disease’ (CVD) as any reported diagnosis of angina, heart attack or coronary heart disease. Respondents of all ages were also asked “(Other than during pregnancy,) have you ever been told by a doctor or health professional that you have diabetes or sugar diabetes. We combined self-reported diagnosed and borderline diabetes as ‘diabetes’ for analysis. Unfortunately fasting blood glucose levels were only available for the participant subset seen in the mornings, and were therefore not used in our analyses.

Data were analyzed for the three original liver enzyme markers associated with BPA in 2003/04: gamma-glutamyl transferase, which was measured by NHANES using an enzymatic rate method; lactate dehydrogenase, using an enzymatic rate method; alkaline phosphatase, using a 2-Amino-2-Methyl-1-Propanol (AMP) buffer. Full details of analyte extraction and measurement are available at http://www.cdc.gov/nchs/nhanes.htm.

### Statistical Analysis

The NHANES survey uses a complex cluster sample design, with certain demographic groups (including those in low socioeconomic positions and Mexican Americans) over-sampled to obtain adequate representation. To account for the complex sampling, weighted estimates were computed, in accordance with the NHANES ‘Analytic and Reporting Guidelines’ (http://www.cdc.gov/nchs/data/nhanes/nhanes_general_guidelines_june_04.pdf [accessed 22nd September 2009]. Sampling errors were estimated by the Taylor series (linearization) method to take account of stratification and clustering using the provided ‘masked variance pseudo-psu’ and ‘pseudo-stratum’ variables. All analyses were conducted using Stata SE Version 10. DM, NR and WH analyzed the data.

The respondents included in our analyses were those aged 18 to 74 years (of those randomly selected by NHANES for BPA measurement) in order to focus on adult health conditions. The 74 year age cut-off was selected to minimize biases caused by co-morbidity and non-representation of seniors in institutions. Six individuals with BPA concentrations greater than 80.1 ng/ml in 2005/06 were excluded from our analyses, as these high levels were outside the range of BPA in the original 2003/04 sample. One respondent was excluded due to a missing urinary creatinine value in 2003/04, resulting in an included sample of n = 1455 respondents in 2003/04 and n = 1493 in 2005/06.

Standardized BPA measures were calculated using single wave, cohort specific z-scores of BPA for independent NHANES wave analyses and pooled wave, cohort specific z-scores of BPA for pooled analyses. Logistic regression models were used to estimate odds ratios of self-reporting doctor diagnosed heart disease and diabetes per one standard deviation increase in BPA concentrations. Linear regression models were used to estimate associations between logged levels of liver enzymes and standardized BPA concentrations.

Regression models were adjusted for potential confounders, including socioeconomic markers which Calafat and colleagues [5] reported to be associated with BPA concentrations, and urinary creatinine to account for urine concentration [27]. Adjustment was for: race/ethnicity, from self-description and categorized into: Mexican American, other Hispanic, non-Hispanic White, non-Hispanic Black, and other race (including multi-racial); education, categorized into: less than high school, high school diploma (including GED), more than high school, and unknown education; annual household income, categorized into: less than $20,000; $20,000 to $35,000; $35,000 to $65,000; over $65,000, and unknown income; smoking (from self-reported status asked in those aged 20 and over), categorized into: never smoked, former smoker, smoking some days, smoking every day, and unknown smoking status; Body Mass Index (‘BMI’, measured weight in kilograms divided by the square of measured height in meters), categorized into: underweight (BMI<18.5), recommended weight (BMI 18.5 to 24.9), overweight (BMI 25.0 to 29.9), obese I (BMI 30.0 to 34.9), obese II (BMI 35.0 or above), and unknown BMI; waist circumference (in quartiles, with a missing value group); and, urinary creatinine concentration in mg/dl.

Generalized additive models with cubic regression splines [28] were used to explore the functional form of the relationships between presence of a cardiovascular disease diagnosis and BPA concentration. These models provide a method of identifying departures from linearity in exposure-response relationships. Linearity was assessed by visual inspection of the estimated spline functions and by consideration of the “estimated degrees of freedom” (edf) for the smoothed BPA term. Values of the edf close to 1 were taken as evidence of linearity. These models were fitted in the statistical software ‘R’ using the mgcv package for generalized additive modeling.

### Power Calculations

Power calculations for analyses of diagnosis presence and liver enzyme concentrations were conducted using the approaches proposed by Hsieh et al [29] for logistic and linear regression respectively. The 2003/04 sample provided 80% power to detect unadjusted Odds Ratios of 1.4 for diagnoses of 5% prevalence per standard deviation change in BPA concentration. Similar calculations for the 2005/06 sample gave a reduced power of 73% to detect an effect size of this magnitude, after allowing for the shift in the BPA distribution between waves. For liver enzymes, the detectable effect size for 80% power was estimated using the standardized regression coefficient of 0.075 found in 2003/04. Power to detect this effect size was reduced to 74% in the 2005/06 sample after allowing for the reduction in variation in BPA exposure levels.

## Results

The study sample included 694 men and 761 women with measured urinary BPA in the 2003/04 cohort, and 720 men and 773 women in 2005/06. The socio-demographic characteristics of the two samples were otherwise similar (Table 1).

**Table 1:** Sample characteristics by NHANES wave.

	NHANES 2003/04	NHANES 2005/06	Test of difference between NHANES years*	Pooled data
	N (% of sample)	N (% of sample)	p-value	N (% of sample)
**Gender**			0.730	
Men	694 (48.2%)	720 (49.1%)		1414 (48.6%)
Women	761 (51.8%)	773 (51%)		1534 (51.4%)
**Age-group (yrs)**			0.617	
18–29	449 (23.5%)	476 (22.9%)		925 (23.2%)
30–39	244 (20.4%)	269 (19.9%)		513 (20.1%)
40–49	252 (22.8%)	250 (22.3%)		502 (22.6%)
50–59	182 (17.7%)	201 (18.5%)		383 (18.1%)
60–74	328 (15.7%)	297 (16.5%)		625 (16.1%)
**Race/ethnicity**			0.650	
Mexican American	324 (8.5%)	312 (8.2%)		636 (8.3%)
Other Hispanic	57 (4.3%)	51 (3.4%)		108 (3.9%)
Non-Hispanic White	690 (69.2%)	669 (70.6%)		1359 (69.9%)
Non-Hispanic Black	313 (11.6%)	396 (12.2%)		709 (11.9%)
Other race (including multi-racial)	71 (6.4%)	65 (5.5%)		136 (6%)
**Level of education**			0.332	
Less than high school diploma	430 (18.1%)	390 (16.1%)		820 (17.1%)
High school diploma (including GED)	356 (25.9%)	383 (24.8%)		739 (25.3%)
Some college education	669 (56.1%)	719 (59%)		1388 (57.6%)
Unknown	n/a	1 (0.1%)		1 (0.06%)
**Household annual income**			0.160	
<$20,000	342 (15.9%)	299 (13%)		641 (14.4%)
$20,000 to $35,000	306 (18.1%)	286 (16.2%)		592 (17.2%)
$35,000 to $65,000	354 (27%)	374 (26.1%)		728 (26.5%)
>$65,000	354 (33.4%)	452 (40.3%)		806 (36.9%)
Unknown	99 (5.6%)	82 (4.4%)		181 (5%)
**Body mass Index (BMI)**			0.526	
Low weight, BMI<18.5	31 (2.1%)	34 (2.7%)		65 (2.4%)
Recommended, BMI 18.5 to 24.9	469 (33.6%)	440 (30.8%)		909 (32.2%)
Overweight, BMI 25.0 to 29.9	448 (30.4%)	486 (31.5%)		934 (30.9%)
Obese I, BMI 30.0 to 34.9	283 (20%)	292 (18.7%)		575 (19.3%)
Obese II, BMI> = 35	199 (12.2%)	231 (15.7%)		430 (14%)
Unknown	25 (1.6%)	10 (0.6%)		35 (1.1%)
**Cigarette smoking**			0.594	
<100 cigarettes in lifetime	640 (48.8%)	715 (48.6%)		1355 (48.7%)
Former smoker	311 (22.6%)	292 (22%)		603 (22.3%)
Some days	63 (4.4%)	48 (3.2%)		111 (3.8%)
Every day	264 (20.5%)	277 (22.7%)		541 (21.7%)
Unknown	177 (3.8%)	161 (3.6%)		338 (3.7%)

* **Note: estimate based on survey weighted age, sex ethnicity adjusted logistic models in adults aged 18–74 years with valid BPA measures, comparing NHANES wave.**

**Figure 1:**
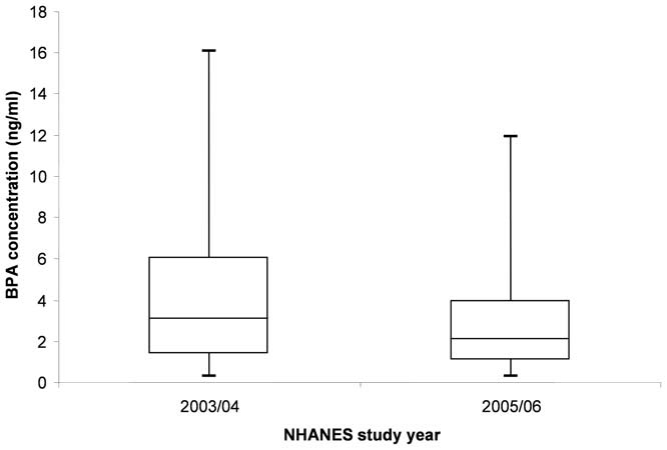
Distribution of Bisphenol A concentration (ng/ml) in NHANES 2003/04 and 2005/06. Note: Boxes represent upper and lower quartiles with median line, whiskers end at 5^th^ percentile (below LLOD) and 95^th^ percentile of distribution. Data from adults aged 18 to 74 years.

Urinary BPA levels were lower in the 2005/06 cohort (Figure 1) than in the 2003/04 cohort: unadjusted geometric means: 1.79 ng/ml (95% CI: 1.64 to 1.96) vs 2.49 ng/ml (CI: 2.20 to 2.83); unadjusted arithmetic means: 3.30 ng/ml (CI: 2.88 to 3.72) vs 4.59 ng/ml (CI: 3.95 to 5.24). In regression analyses of logged BPA concentration adjusting for age, gender, ethnicity and urinary creatinine, the difference in BPA levels between NHANES waves was significant: (back transformed beta = −1.33 ng/ml, 95% CI: −1.19 to −1.50, p = 0.00002).

Per standard deviation increases in BPA concentration were associated with positive responses to questions about physician diagnoses of myocardial infarction in age, sex and ethnicity adjusted models in 2005/06 (OR = 1.31, 95%CI: 1.02 to 1.68, p = 0.036), with OR estimates for reported angina and ‘coronary heart disease’ being similar to 2003/04 but narrowly missing conventional two sided statistical significance (Table 2). In pooled models, positive responses to any of the three questions above (here termed ‘cardiovascular diseases’) was associated with BPA concentrations (per SD increase in BPA OR = 1.26, CI: 1.11 to 1.43, p = 0.001). The estimated associations of BPA with reported diabetes in 2005/06 was modest but with wide confidence intervals; however, the diabetes association remained significant in pooled data (OR = 1.27, CI: 1.12 to 1.43, p = 0.0004).

**Table 2:** Disease prevalence, and survey weighted, age, gender and ethnicity adjusted model estimates (odds ratios with 95% confidence intervals) of associations with per standard deviation increases of Bisphenol A concentration with adjustment for urinary creatinine: adults aged 18 to 74.

	NHANES 2003/04	NHANES 2005/06	Pooled data
Condition	N (%)	OR (95% CI), p-value	N (%)	OR (95% CI), p-value	N (%)	OR (95% CI), p-value
Myocardial Infarction	42/1277 (2.8)	**1.34 (1.11 to 1.61), p = 0.004**	38/1329 (3.2)	**1.31 (1.02 to 1.68), p = 0.036**	80/2606 (3.0)	**1.28 (1.13 to 1.46), p = 0.001**
Angina	42/1274 (2.7)	**1.26 (1.08 to 1.46), p = 0.006**	31/1327 (2.4)	1.29 (0.98 to 1.70), p = 0.064	73/2602 (2.5)	**1.27 (1.10 to 1.47), p = 0.002**
Coronary Heart Disease	46/1276 (2.8)	**1.45 (1.16 to 1.80), p = 0.003**	42/1328 (3.3)	1.29 (0.96 to 1.74), p = 0.089	88/2605 (3.1)	**1.37 (1.16 to 1.63), p = 0.001**
CVD (any diagnoses of MI, angina or CHD)	83/1279 (5.0)	**1.31 (1.10 to 1.56), p = 0.005**	76/1331 (5.9)	1.21 (0.92 to 1.59), p = 0.164	159/2610 (5.5)	**1.26 (1.11 to 1.43), p = 0.001**
Diabetes	136/1456 (7.9)	**1. 41 (1.21 to 1.64), p = 0.0003**	141/1491 (8.8)	1.07 (0.85 to 1.34), p = 0.536	277/2947 (8.4)	**1.27 (1.12 to 1.43), p = 0.0004**

Odds ratios of reporting heart disease and diabetes diagnoses were computed in fully adjusted models (Table 3), using z-scores of BPA and adjusting for age, sex, race/ethnicity, education, income, smoking, BMI, waist circumference and urinary creatinine concentrations. Per standard deviation increases in BPA concentration were associated with coronary heart disease (OR = 1.33, CI: 1.01 to 1.75, p = 0.043) in 2005/06 but narrowly missed two sided significance for myocardial infarction in 2005/06 (OR = 1.39, CI: 1.00 to 1.94, p = 0.051). Associations for any of the three reported responses to cardiovascular disease were present in pooled data in the fully adjusted models (OR = 1.26, CI: 1.10 to 1.44, p = 0.001). There was no association between BPA concentration and diabetes in fully adjusted models in 2005/06 (OR = 1.02, CI: 0.76 to 1.38, p = 0.872), but an overall association was still present in pooled data (OR = 1.24, CI: 1.10 to 1.40, p = 0.001).

**Table 3:** Fully adjusted* survey weighted model estimates (odds ratios with 95% confidence intervals) of disease associations per standard deviation increase of Bisphenol A concentration: adults aged 18 to 74.

	NHANES 2003/04	NHANES 2005/06	Pooled data
Condition	OR (95% CI), p-value	OR (95% CI), p-value	OR (95% CI), p-value
Myocardial Infarction	**1.40 (1.07 to 1.84), p = 0.017**	1.39 (1.00 to 1.94), p = 0.051	**1.32 (1.15 to 1.52), p = 0.0003**
Angina	**1.27 (1.06 to 1.54), p = 0.015**	1.16 (0.88 to 1.53), p = 0.262	**1.24 (1.07 to 1.43), p = 0.005**
Coronary Heart Disease	**1.60 (1.11 to 2.32), p = 0.016**	**1.33 (1.01 to 1.75), p = 0.043**	**1.42 (1.17 to 1.72), p = 0.001**
CVD (any diagnoses of MI, angina or CHD)	**1.34 (1.10 to 1.66), p = 0.008**	1.18 (0.88 to 1.59), p = 0.243	**1.26 (1.10 to 1.44), p = 0.001**
Diabetes	**1.40 (1.25 to 1.56), p = 0.00001**	1.02 (0.76 to 1.38), p = 0.872	**1.24 (1.10 to 1.40), p = 0.001**

* models adjusted for age, gender, ethnicity, education, income, BMI, waist circumference, smoking status and urinary creatinine.

The association between BPA and the three liver enzymes previously examined were not statistically significant in age, sex and ethnicity adjusted models (Table 4) or fully adjusted models (Table 5) in 2005/06, although overall associations in the pooled data were still present for alkaline phosphatase (logged ALP association with per-SD increase in BPA concentration beta = 0.02, CI: 0.01 to 0.03, p = 0.002) and lactate dehydrogenase (beta = 0.01, CI: 0.003 to 0.02, p = 0.009).

**Table 4:** Serum liver enzyme concentrations (geometric means) and survey weighted, age, gender and ethnicity adjusted model estimates† (with 95% confidence intervals) of associations between logged liver enzymes per standard deviation increase of Bisphenol A concentration, with adjustment for urinary creatinine: adults aged 18 to 74.

	NHANES 2003/04	NHANES 2005/06	Pooled data
Liver enzymes	N	geometric mean 95% CI	beta (95% CI), p-value	N	geometric mean 95% CI	beta (95% CI), p-value	N	geometric mean 95% CI	beta (95% CI), p-value
Gamma-glutamyl transferase (u/l)	1377	21.1 (20.1 to 22.2)	**0.08 (0.03 to 0.12), p = 0.0002**	1402	20.8 (19.7 to 22.0)	−0.04 (−0.08 to 0.01), p = 0.131	2779	21.0 (20.2 to 21.7)	0.02 (−0.02 to 0.06), p = 0.269
Alkaline phosphatase (u/l)	1378	64.2 (62.9 to 65.5)	**0.03 (0.02 to 0.05), p = 0.001**	1402	65.8 (64.0 to 67.7)	0.02 (−0.004 to 0.04), p = 0.094	2780	65.0 (63.9 to 66.1)	**0.03 (0.01 to 0.04), p = 0.001**
Lactate dehydrogenase (u/l)	1374	123.7 (121.0 to 126.4)	**0.02 (0.01 to 0.03), p = 0.006**	1378	123.3 (121.6 to 125.1)	0.01 (−0.004 to 0.02), p = 0.147	2752	123.5 (122.0 to 125.1)	**0.01 (0.01 to 0.02), p = 0.002**

† Coefficients represent change in logged analyte level for each standard deviation change in bisphenol A.

**Table 5:** Fully adjusted* survey weighted model estimates† (with 95% confidence intervals) of logged liver enzyme analyte associations per standard deviation increase of Bisphenol A, with adjustment for urinary creatinine: adults aged 18 to 74.

	NHANES 2003/04	NHANES 2005/06	Pooled data
Liver enzymes	beta (95% CI), p-value	beta (95% CI), p-value	OR (95% CI), p-value
Gamma-glutamyl transferase (u/l)	**0.06 (0.03 to 0.10), p = 0.002**	−0.03 (−0.07 to 0.01), p = 0.133	0.01 (−0.02 to 0.05), p = 0.380
Alkaline phosphatase (u/l)	**0.02 (0.01 to 0.04), p = 0.008**	0.02 (−0.003 to 0.04), p = 0.094	**0.02 (0.01 to 0.03), p = 0.002**
Lactate dehydrogenase (u/l)	**0.01 (0.001 to 0.03), p = 0.036**	0.01 (−0.003 to 0.02), p = 0.135	**0.01 (0.003 to 0.02), p = 0.009**

* models adjusted for age, gender, ethnicity, education, income, BMI, waist circumference, smoking status and urinary creatinine.

† Coefficients represent change in logged analyte level for each standard deviation change in bisphenol A.

**Figure 2:**
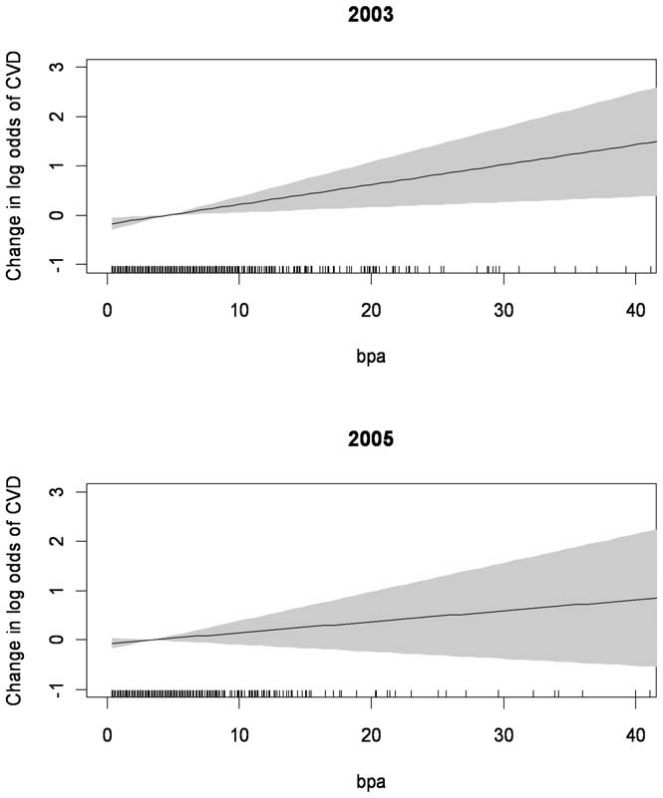
Dose response curves for the association of BPA concentration with logged odds of Cardiovascular disease diagnoses. Generalized additive models with cubic regression splines in NHANES 2003/04 and 2005/06.

Models of the shape of the dose response curve (generalized additive models with cubic regression splines, to 4 standard deviations above mean of BPA concentration, Figure 2) showed that there were linear relationships between BPA concentration and cardiovascular disease in both 2003/04 and in 2005/06.

As a post hoc analysis, we examined associations between BPA concentration and the other diseases originally examined in the 2003/04 data [23], namely arthritis, asthma, cancer, chronic bronchitis, emphysema, liver disease, stroke or thyroid disease. We found no associations between BPA concentrations and other diseases in the 2005/06 cohort or in pooled analyses (data available from authors).

## Discussion

Previously, we reported an analysis of NHANES 2003/04, in which we found associations between raised urinary BPA concentrations and coronary heart disease, diabetes and raised liver enzymes in adults. There was no evidence of association with other reported health outcomes, suggesting specificity of the findings. Here we sought to determine if these associations were present again in the NHANES 2005/06 sample, and to calculate pooled estimates of the effect sizes. These analyses use the only large-scale (and high-quality) population-representative datasets available. Urinary BPA concentrations were lower in 2005/06 than in the earlier NHANES wave, with a geometric mean value of 1.79 compared with the earlier mean of 2.49. This represents a fall of around 30%. After adjusting for potential confounders, we found that higher BPA concentrations were again associated with heart disease diagnoses. Associations of BPA concentration with diabetes and liver enzymes were not statistically significant in the 2005/06 data, although pooled estimates remained significant.

Cardiovascular disease results from a complex interaction of genetic, lifestyle and environmental factors. Whilst several personal risk factors for developing heart disease have been identified, including smoking, diabetes and dyslipidemia, the contribution of environmental contaminants has received comparatively less attention. To date, the most widely reported associations between cardiovascular disease and pollutants have centred on exposure to air particulates, heavy metals, notably arsenic and lead, and persistent organic pollutants.

It is pertinent to consider what is known of these previously reported associations, to place the current findings of associations with BPA into context. Studies of over 100 million people across the USA and Europe have shown that for every 10 mg/m^3^ increase in aerial fine particulate matter (PM10), there is a 0.3%–0.7% increase in cardiovascular mortality [30], [31]. These effects can appear and disappear quickly; transient increases in very fine particulate matter are associated with an increased incidence of acute myocardial infarction within a few hours, highlighting the high sensitivity of cardiovascular tissues to the effects of environmental contaminants [32]. Such associations are plausible through several biological mechanisms, including alterations to vasomotor tone [33], [34], thrombogenic effects [35] and the induction of systemic inflammation [36]. In the case of arsenic, altered vascular tone appears to be of more importance to the elevation in cardiovascular risk than vascular inflammation or dislipidemia [37], [38]. Animal studies have implicated dysregulation of nitric oxide production and reactivity in arsenic's adverse effects on cardiovascular risk [37], potentially via oxidation of protein thiol groups, including endothelial nitric oxide synthase (eNOS) [39]. A similar mechanism has been suggested for lead, which has been associated with hypertension in both animal and human epidemiology studies [40]. Both polychlorinated biphenyls (PCBs) and 2,3,7,8-tetrachlorodibenzo-*p*-dioxin (TCDD) can induce oxidative stress-mediated alterations to normal vascular endothelial cell function, leading to pro-inflammatory changes [41], [42]. TCDDs can in addition cause an increase in atherogenic serum lipid levels in animals and humans and a decrease in low density lipoprotein receptors in the liver [43], [44]. Such a combination of damage to endothelial cells with elevation in serum lipid levels could be expected to increase cardiovascular risk.

The relevance of these mechanisms to the reported association between BPA and cardiovascular disease in the current study remains subjective. For example, inflammation is a dynamic response of vascularised tissues to injury, whilst excessive or prolonged inflammation is associated with various diseases in addition to cardiovascular disease [45]. Following oral ingestion, BPA is not considered a persistent compound, but neither is it immediately cleared from the body, and there appears to be virtually continuous human exposure [46]. It is lipophilic (the log of the octanol-water partition co-efficient (K_ow_) for BPA is between 2.2 and 3.82 (NTP, 2008)) and it is plausible that partitioning to lipid rich tissues could occur with frequent exposure. This possibility is supported by recent findings that the population-based half life for BPA is significantly longer than the previous estimates of around 6 hours [3]. The metabolism of BPA is reported to induce oxidative stress in rat hepatocytes following long term (30 day) oral intake [10] and oxidative cellular damage has been reported in a number of other experimental contexts [11], [12], [13], [14]. Slow release of BPA or metabolites from tissue sites could theoretically lead to oxidative endothelial cell damage.

Urinary BPA concentrations have previously been shown to be positively associated with oxidative stress markers. Hong et al [47] found a significant dose-responsive relationship between BPA and the oxidative stress markers malondialdehyde (MDA) and 8-deoxyguanosine (8-OHG) in a study of 960 adults, although the relationship was not present in multiple regression models adjusted for confounders including age and sex. Positive associations were also reported between urinary BPA, MDA and 8-OHD, and with C-reactive protein, an inflammatory marker, in a cross-sectional study of 134 post-menopausal women, but not in men or pre-menopausal women from the same study [48]. These gender differences suggest that occupancy and activation of the estrogen receptor may have been a contributing factor in the induction of oxidative stress.

BPA is believed to exert its biological effects largely through loose occupancy of the estrogen receptor, and there are reports of both estrogen agonist [7] and androgen antagonist [8] activity. BPA also binds strongly to the estrogen-related receptor-gamma (ERR-gamma), the function of which is unknown [9]. BPA exposure of cell suspensions results in lipid accumulation in adipocytes and hepatoma cell lines [49]. A variety of other effects of BPA have been noted, including disrupted pancreatic Beta-cell function producing insulin resistance in mice exposed to oral BPA doses well below the current lowest observed adverse effect level considered by the US-EPA [15]. Two days of low-dose BPA injections also produced insulin resistance in mice [50]. Associations have been described previously between environmental toxins, body weight, and diabetes [8], [51] leading to suggestions that exposure to certain environmental pollutants may initiate or exacerbate the development of obesity [17] and associated health problems [33]. Diabetes and dyslipidemia are recognized as personal risk factors for developing heart disease. However, associations with diabetes seen in the 2003/04 NHANES survey was not evident in the current study, possibly because of the reduction in statistical power due to the lower levels of BPA in 2005/06, resulting in wide confidence intervals around estimates.

It is possible that an as yet unidentified mechanism could be involved in the effects of BPA on cardiovascular function. The peroxisome proliferation activated receptor gamma, PPARγ plays a central role in the control of energy balance and lipid homeostasis [52] and PPARγ agonists such as rosiglitazone are used to treat insulin sensitivity. There have been reports that in addition to PPARγ-mediated genome effects on genes important for maintaining vascular tone (including eNOS), PPARγ agonists may activate or inhibit ion channel activity in vessel walls directly. In studies of isolated vascular smooth muscle, rosiglitazone was able to attenuate inward calcium currents and enhance calcium-activated potassium currents [53]. Certain BPA derivatives, including bisphenol A diglycidyl ether (BADGE) are peroxisome proliferation activated receptor gamma (PPARγ) antagonists and BADGE can block the adipogenic action of the receptor [54]. Although BPA has been reported to increase adipocyte differentiation, it does not appear to affect PPARγ through the same mechanism as BADGE. Additional studies are clearly called for to examine in detail the relationship between BPA and its derivates and PPARγ signaling.

A major limitation of our original analysis on NHANES 2003/04 [23] was that no replication data were available to help exclude the possibility of a chance finding. The 2005/06 analyses presented here are based on an entirely new population sample, providing an opportunity for independent replication of the earlier findings. BPA concentrations in 2005/06 were substantially lower than in 2003/04, resulting in a reduced power to detect real associations. Despite this, we found near or significant associations with responses to the three questions about heart disease in NHANES 2005/06, and highly significant p-values for the pooled estimates of association. We can now conclude that chance is an implausible explanation for the BPA association with heart disease. Replication of the BPA associations with diabetes and liver enzymes are less certain, with estimates for 2005/06 having wide confidence intervals and not reaching statistical significance. Because of the lower BPA concentration levels in 2005/06 a reduction in the strengths of association is to be expected. The BPA measures in NHANES are based on single spot specimens, so misclassification from this single snapshot of body burden will have resulted in a smaller (diluted) estimate of the strength of association between BPA and the conditions of interest: the true associations are likely to be much stronger. In addition, the pooled estimates of association suggest that the new data is consistent with the original findings: larger samples will be needed to provide accurate estimates of BPA associations with diabetes or liver enzymes to exclude the possibility of real associations being present.

The cross sectional nature of the associations reported need to be treated with caution, as it is theoretically possible, for example, that those with cardiovascular disease change their diets in such a way as to increase BPA exposure. In addition, it is possible that BPA exposure is associated with differential survival in cardiovascular disease. Longitudinal data demonstrating that high BPA concentrations predict later onsets of heart disease or diabetes would strengthen the evidence for BPA playing a causal role. More work is also needed to understand the mechanisms of effect underlying the BPA exposure heart disease association, including *in vitro* and *in vivo* studies.

### Conclusions

Urinary BPA concentrations in the NHANES adult population representative sample of the United States in 2005/06 were substantially lower than in 2003/04. Despite this, we have replicated earlier findings that higher urinary concentrations of Bisphenol A are associated with an increased prevalence of coronary heart disease.

Associations between urinary BPA concentration and diabetes or liver enzyme increases were not statistically significant in 2005/06, but confidence intervals were wide and associations remained in pooled data. Detailed studies are needed to clarify the mechanisms explaining the statistical association between BPA and adult morbidity.
